# Molecular insights into the bioactivity of H-thiazine compounds against breast cancer cells: a computational study

**DOI:** 10.1007/s40203-025-00542-y

**Published:** 2026-01-14

**Authors:** Lesego M. Mogoane, Vincent A. Obakachi, Penny P. Govender, Krishna K. Govender

**Affiliations:** Department of Chemical Sciences, University of Johannesburg, Doornfontein Campus, P.O. Box 17011, Johannesburg, 2028 South Africa

**Keywords:** Breast cancer, EGFR, Thiazine derivatives, Computational drug design, Molecular dynamics

## Abstract

Breast cancer, a leading cause of global mortality, necessitates novel therapies targeting key drivers like the epidermal growth factor receptor (EGFR). This computational study evaluates nine 4-phenyl-2H-[1,3]thiazino[3,2-a]benzimidazol-2-imine (H-thiazine) derivatives as potential EGFR inhibitors. Using molecular docking, ADMET profiling, molecular dynamics simulations, and binding energy calculations, we identified methyl- and bromine-substituted derivatives as probable candidates that outperform the reference drug Olmutinib in terms of binding affinity, pharmacokinetics, and stability. Although these compounds showed promising bioactivity, in silico toxicity screening indicated potential AMES mutagenicity and hERG-II inhibition, highlighting important safety liabilities. Overall, thiazine derivatives represent viable scaffolds for EGFR-targeted anti-cancer development; however, further optimization and experimental validation, including biochemical assays and genotoxicity testing, are required to confirm their therapeutic potential.

**Supplementary Information:**

The online version contains supplementary material available at 10.1007/s40203-025-00542-y.

## Introduction

Breast cancer is a critical threat to global health, with 2.3 million new diagnoses and 670,000 deaths reported worldwide in 2022(World Health Organization 2022). In Sub-Saharan Africa, 146,130 cases and 71,662 deaths were recorded, with projections indicating an 85.7% increase in new cases and a 89% rise in deaths by 2040(Sung et al. 2021). Current treatments, including surgery, chemotherapy, radiotherapy, hormone therapy, and targeted therapies, often cause severe side effects such as hair loss, nausea, fatigue, and increased infection risk, as well as long-term complications like heart problems and secondary cancers (National Cancer Institute 2023). Resistance to these therapies further limits their effectiveness, highlighting the need for more targeted and efficient treatment options developed for a specific breast cancer subtype (Waks and Winer 2019).

**Fig. 1:**
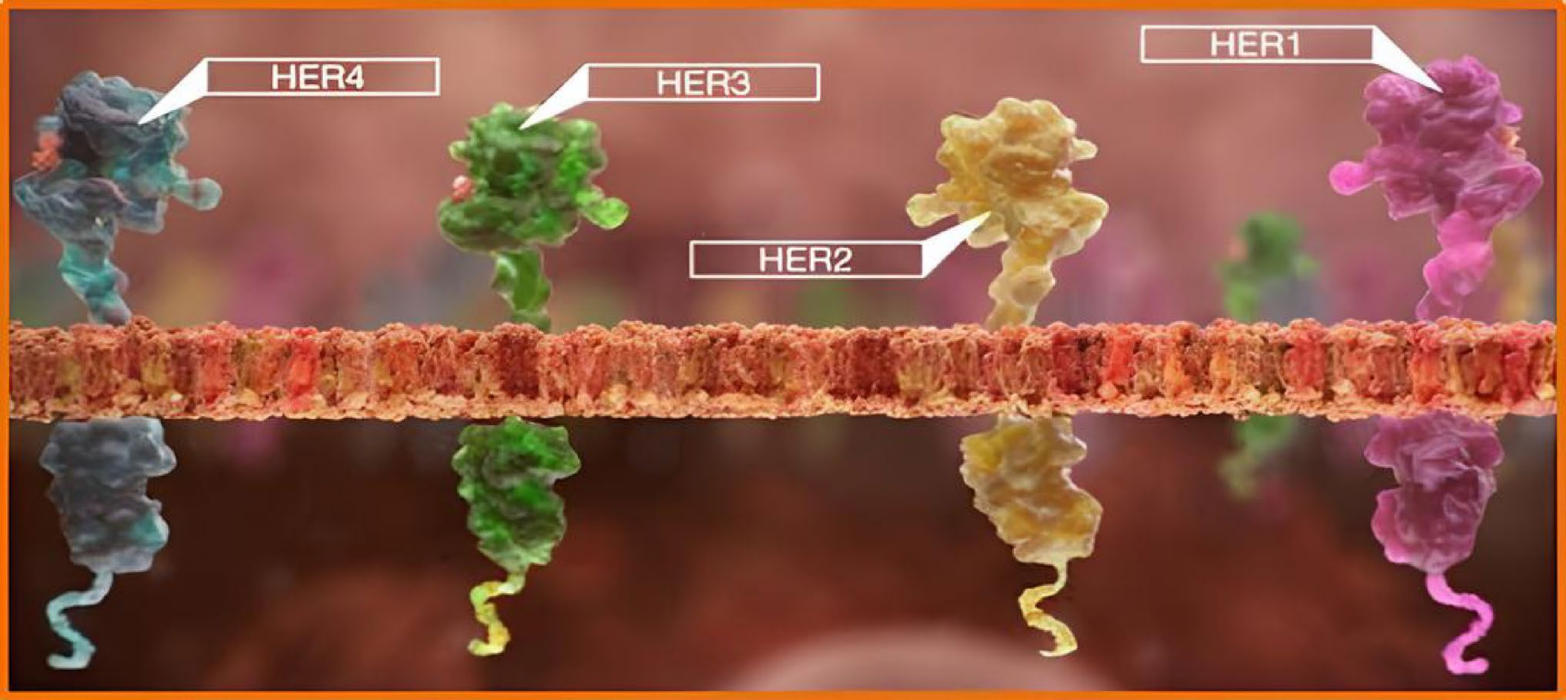
ErbB (HER) family (Ismail et al. 2019)

A promising strategy involves targeting molecular drivers like the epidermal growth factor receptor (EGFR), a member of the Epidermal growth factor receptor (ErbB) family of receptor tyrosine kinases, which is overexpressed in aggressive breast cancers and associated with poor clinical outcomes (Yarden and Sliwkowski 2001; Masuda et al. 2012). EGFR, along with HER2, HER3, and HER4, features an extracellular domain, a transmembrane segment, and an intracellular tyrosine kinase domain that regulates cell growth and survival, as shown in Fig. 1 (Hynes and Lane 2005; Berasain et al. 2009). Inhibitors such as gefitinib, cetuximab, and lapatinib target EGFR but face challenges including resistance and suboptimal pharmacokinetics (Baselga and Arteaga 2005; Tebbutt et al. 2013). Developing next-generation EGFR inhibitors with improved selectivity and pharmacokinetics (Sharma et al. 2007) is crucial for enhancing efficacy and reducing adverse effects.

**Fig. 2:**
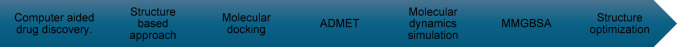
In Silico approaches to drug discovery and development

Computational tools, particularly structure-based drug design (SBDD), facilitate drug discovery by leveraging 3D protein structures to design potent and selective compounds (Anderson 2003; Kalyaanamoorthy & Chen 2011). These tools, as shown in Fig. 2, are critical for identifying novel chemical scaffolds with therapeutic potential, particularly thiazine compounds.

Thiazines, a class of heterocyclic molecules found in natural and synthetic compounds, exhibit diverse biological activities, including antibacterial, anti-cancer, anti-inflammatory, antitumor, and antiviral properties (Tilak 1960; Sanphanya et al. 2013; Sharma and Makkar 2016; Badshah & Naeem 2016). Sulphur and nitrogen-containing heterocycles have been widely studied for their anti-cancer, antimicrobial, and anti-inflammatory properties, attributed to their ability to engage in diverse hydrogen bonding and π–π stacking interactions within enzyme active sites (Kumar et al. 2024; Wang et al. 2021; Hamad 2025). Despite their promise, thiazine derivatives remain underexplored as EGFR inhibitors for breast cancer.

The parent scaffold 4-phenyl-2H-[1,3]thiazino[3,2-a]benzimidazol-2-imine (H-thiazine) offers an attractive foundation for rational optimization, particularly through small substituents such as methyl and halogens that can modulate electronic distribution, lipophilicity, metabolic stability, and binding affinity (Chiodi & Ishihara 2024; Shi et al. 2022). These substituents have been shown to enhance receptor interactions while maintaining drug-like molecular properties. Given the chemical flexibility and pharmacological potential of the H-thiazine scaffold, a systematic computational evaluation is warranted.

**Fig. 3:**
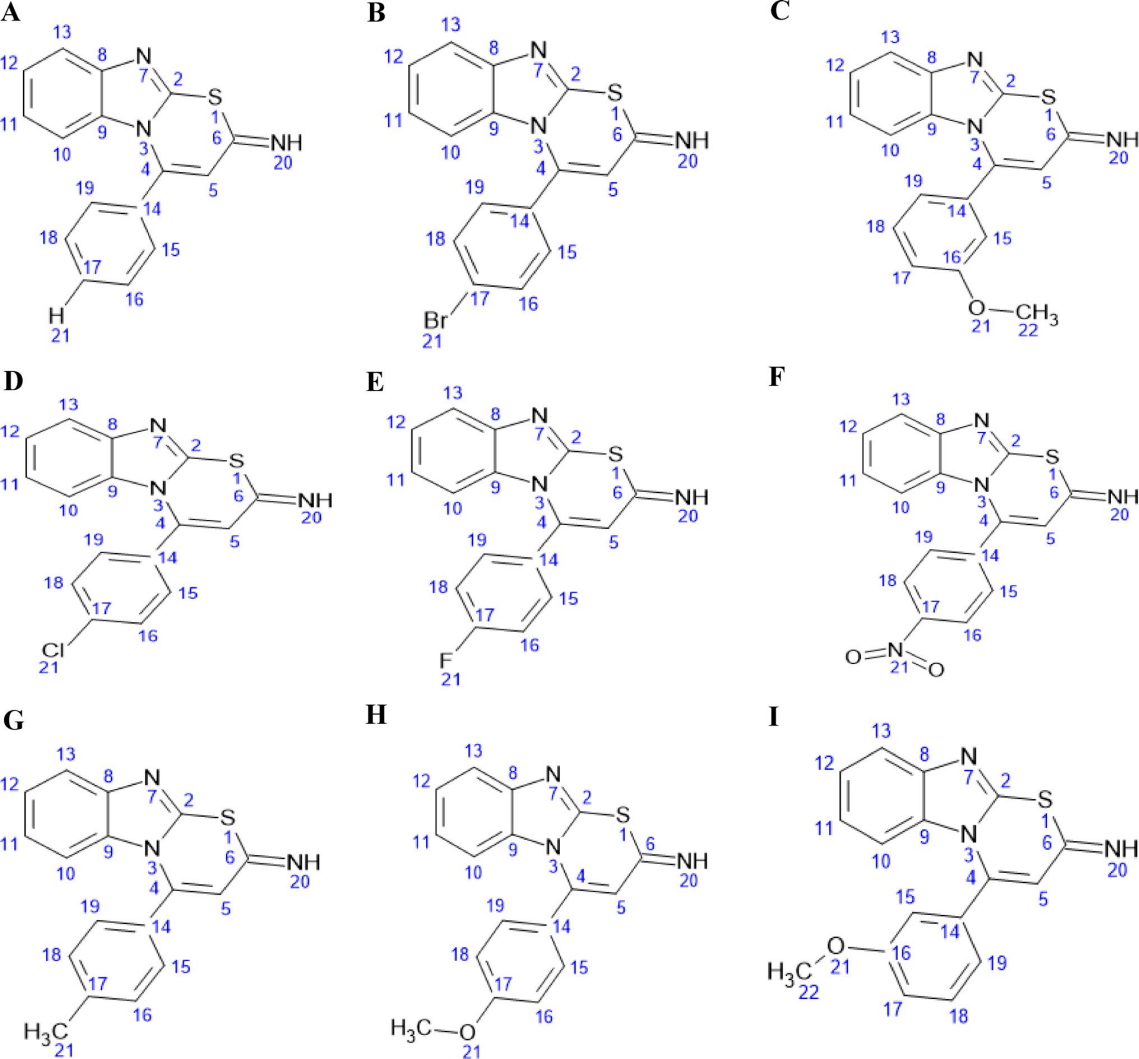
Chemical structures of H thiazine and its derivatives (**A**–**I**). **A** Compound H, **B** Compound Br, **C** Compound M-M1, **D** Compound Cl, **E** Compound F, **F** Compound Nitro, **G** Compound Methyl, **H** Compound P-methoxy, and **I** Compound M-M2. Substituent positions for each derivative are indicated at C17 for compounds H, Br, Methyl, Cl, F, P-methoxy, Nitro, and C16 for compounds M-M1 and M-M2

In this study, nine thiazine derivatives (Fig. 3) were evaluated, including the parent H-thiazine scaffold, as potential EGFR inhibitors. Using in silico approaches, including molecular docking, ADMET profiling, molecular dynamics simulations, and MM-GBSA binding free energy calculations, this study assesses the binding affinity, pharmacokinetic properties, dynamic stability, and therapeutic potential of these compounds against EGFR in breast cancer. This work presents the first comprehensive computational assessment of thiazine-based derivatives against EGFR, highlighting promising candidates for further optimization and future experimental validation.

## Methods

### Ligand preparation

This study investigated nine thiazine-based derivatives, including 4-phenyl-2H-[1,3]thiazino[3,2-a]benzimidazol-2-imine (H-thiazine), which were optimized at the DFT/M06-2X level with the aug-cc-pVTZ basis set to ensure geometric and electronic stability prior to further analysis (Kendall et al. 1992; Zhao and Truhlar 2008). Olmutinib, an FDA-approved EGFR inhibitor, was included as the reference compound, with its 3D structure retrieved from the PubChem database (PubChem CID: 71461008) (Kim et al. 2023). All ligands were prepared using the LigPrep module in Maestro 13.6 version of the Schrödinger Suite 2023-2, involving hydrogen atom addition and geometry optimization using the OPLS-2005 force field (Banks et al. 2005; Schrödinger 2023a). During ligand preparation (LigPrep), tautomer generation was limited to the most likely tautomer for each compound. While multiple tautomers are chemically possible, the focus was on the dominant physiological form to maintain consistency and reduce computational redundancy. The ligand protonation states were set to pH 7.0 ± 2.0 using Epik 2 (Obakachi et al. 2025). The protonation states were set based on physiological pH, and the predicted pKa values of functional groups were used to justify these assignments. The processed structures were saved for molecular docking calculations.

### Protein preparation

The EGFR tyrosine kinase domain crystal structure (PDB ID: 1M17) was retrieved from the Protein Data Bank (RCSB Protein Data Bank 2024) and prepared using Schrödinger's Maestro suite (Baby et al. 2020; Schrödinger 2023a; Shri et al. 2024). The protein preparation involved removing water molecules and heteroatoms, followed by the addition of hydrogen atoms to the protein structure, and optimizing the structure (Bhargavi et al. 2025). After protein preparation, the structure was split into protein, ligand, and water using the Split Protein function in Maestro. This step ensured that all non-protein components, such as water molecules and the co-crystallized ligand, were separated into independent entries within the workspace navigator. This allowed precise selection of components during receptor grid generation. After this step, the prepared protein structure was finalized and ready for grid generation and docking.

### Molecular docking

Ligand docking is a computational process that predicts how a ligand (a small molecule) binds to a protein (receptor) and estimates their interactions (Wani et al. 2025). The EGFR binding site was defined based on the co-crystallized ligand (erlotinib), with default box dimensions ensuring full coverage of the active site. A 3D receptor grid was then generated around the active site to define the docking region. The grid box encompassed all key catalytic residues to ensure accurate placement of the ligand within the binding pocket. This grid limits the search space to the relevant area, focusing docking simulations on potential interaction sites (Ottu et al. 2025). The Glide XP docking methodology, known for its high precision, was employed to investigate ligand–protein interactions (Naidoo et al. 2025). The OPLS-2005 force field refined docking by accounting for ligand movements (Gunasekaran and Dhakshinamurthy 2024). For each ligand, up to 5 poses were generated and ranked using Glide XP scoring. The docked conformers were evaluated using the docking score, providing valuable insights for future drug design.

### ADME studies

The docked ligands underwent ADME analysis using QikProp in Maestro software to predict pharmacokinetic properties and assess drug-likeness (Schrödinger Release 2023a, b-2 2023a). The analysis was run in fast mode and included identifying the five most similar known drug molecules for comparison (Ali and Al-Hamashi 2024).

### Molecular dynamics simulation

Molecular dynamics (MD) simulations were performed on the EGFR-ligand complexes (PDB ID: 1M17) using the Desmond MD simulation package within the Schrödinger 2023a, b-2 suite, with the OPLS-2005 force field (Schrödinger Release 2023a, b-2, 2023b). The simulations were carried out for selected thiazine derivatives and the reference compound Olmutinib to evaluate the dynamic stability of the protein–ligand interactions (Alzain et al. 2025). Each protein–ligand complex was placed in an orthorhombic simulation box with a 10 Å buffer zone between the solute and the box edges, and solvated using the TIP3P water model. Na⁺/Cl⁻ ions were added to neutralize the system and achieve a physiological 0.15 M ionic concentration, resulting in approximately 47,559–50,615 atoms per system. The OPLS-2005 force field was used for the protein, and ligand parameters were derived automatically using the OPLS-2005 force field. All bond lengths were unconstrained. Coulombic interactions were used for electrostatics, and a 9.0 Å cutoff was applied for nonbonded interactions. The systems were energy-minimized using the steepest descent algorithm, followed by equilibration and production in the NPT ensemble at 300 K and 1 atm for 200 ns. A Nose–Hoover thermostat and a Martyna-Tobias-Klein barostat were used with default relaxation times. No positional restraints were applied. Trajectory data were saved at 20 ps intervals, resulting in 2000 frames per simulation for post-simulation analysis (Gheidari et al. 2024).

### Binding free energy calculations

The binding free energy (ΔG_bind_) is a more reliable criterion for ranking ligands based on their true binding affinity compared to empirical docking scores, such as the XP docking score. It reflects a combination of molecular mechanical energy and solvation contributions (both polar and nonpolar), providing a more physically realistic estimate of ligand-receptor interaction strength (Abbasi et al. 2024). In this study, MM-GBSA calculations were conducted using a custom Python script (thermal_mmgbsa.py) executed via the MobaXterm software (Yazdani et al. 2024). The MM-GBSA was performed on snapshots extracted from 200 ns of the MD simulations (Fereidounpour et al. 2024). The trajectory files were first opened and visually inspected in Maestro to ensure the protein–ligand complex was equilibrated and to determine the total number of frames. Snapshots from frames 1 to 2000 were extracted at an interval of 10. The script was submitted via the terminal. After the calculations were completed, the output files were opened and read to obtain the binding energy results (Mbayo et al. 2025). The average ΔG_bind_ was recorded in kcal/mol, and more negative ΔG_bind values indicate stronger binding. This approach allowed automated processing of molecular dynamics trajectories to compute binding free energies for each ligand–EGFR complex.

### Toxicological profile

To assess the safety of the lead compounds, an in silico toxicology analysis was conducted using the pkCSM web server (PKCSM). The SMILES notation for each compound was used to predict potential toxicity (Dulsat et al. 2023).

## Results and discussion

### Validation of docking protocol

**Fig. 4:**
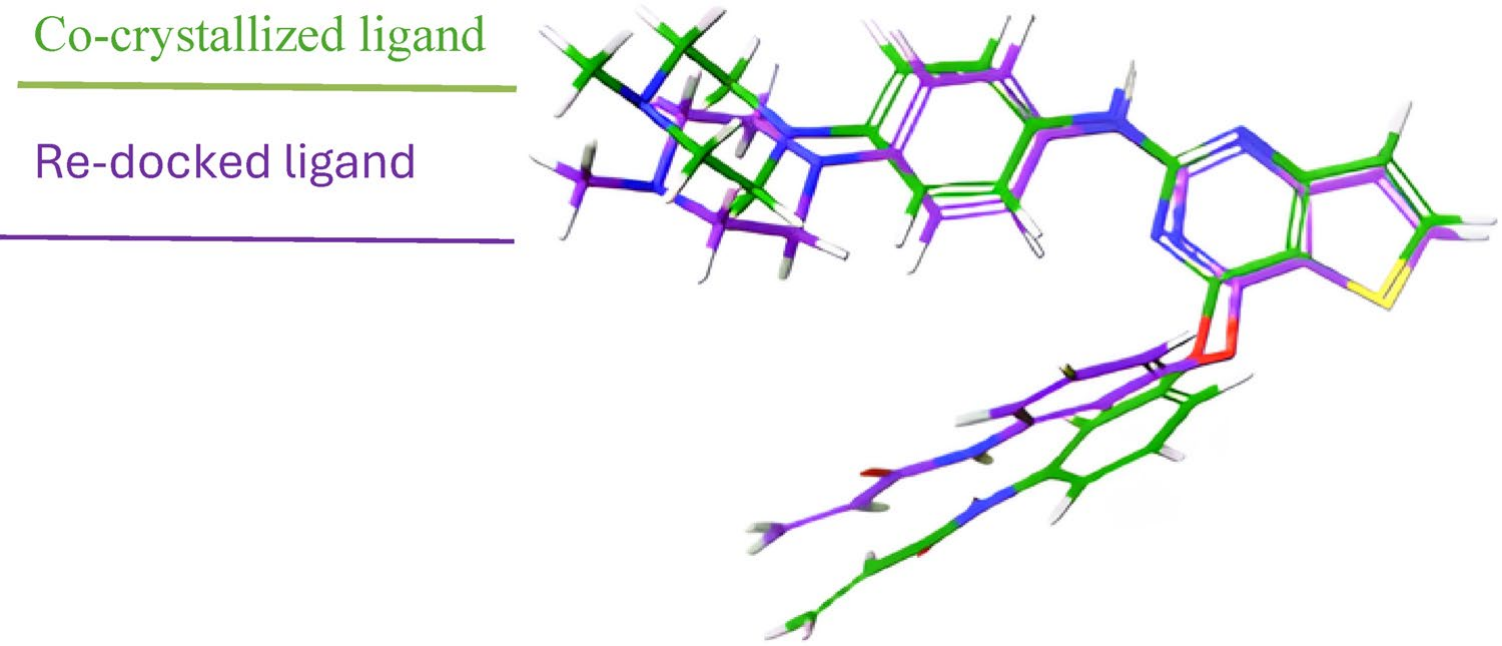
Superimposed structures of co-crystallized and re-docked reference ligand

The accuracy of the molecular docking methodology was rigorously validated by re-docking the co-crystallized ligand within the EGFR active site and comparing its predicted conformation to the original crystal structure, as illustrated in Fig. 4 with the RMSD calculated based on the heavy-atom alignment between the re-docked pose and the crystallographic ligand. The Root Mean Square Deviation (RMSD) between these conformations was determined to be 1.61 Å, a value comfortably below the widely accepted threshold of 2.00 Å (Mukherjee et al. 2010; Zev et al. 2021; Llop-Peiró et al. 2024). This low RMSD reflects a high degree of congruence between the computational predictions and experimental data, affirming the docking protocol's precision in modeling ligand-receptor interactions. In line with this, Zare et al. (2023) reported low RMSD values for 6-bromoquinazoline derivatives, indicating accurate reproduction of experimental ligand poses. This validation is pivotal, as it bolsters confidence in the reliability of predicted binding poses for novel ligands, ensuring alignment with empirical observations and enhancing the robustness of subsequent docking studies for drug discovery applications (Meng et al. 2011).

### Molecular docking and binding affinity analysis

To assess the therapeutic potential of nine thiazine derivatives as EGFR inhibitors, molecular docking and MM-GBSA binding free energy calculations were conducted, with olmutinib, a well-established FDA-approved inhibitor, serving as the reference standard (Elsebaie et al. 2024). All binding free energy (ΔG_bind) values were reported in kcal·mol⁻^1^ for consistency, using the standard conversion factor (1 kcal/mol = − 4.184 kJ/mol).

The results, detailed in Table 1, reveal that olmutinib achieved a docking score of − 4.93 kcal/mol and a binding free energy (ΔG_bind_) of -12.13 kcal/mol, providing a baseline for comparison. Among the thiazine derivatives, the methyl-substituted compound emerged as a standout, exhibiting the most favourable docking score of − 6.95 kcal/mol and a ΔG_bind_ of − 12.86 kcal/mol, indicating superior binding affinity and structural complementarity with the EGFR active site. Similarly, the nitro derivative demonstrated exceptional performance, with a docking score of − 6.32 kcal/mol and the most negative ΔG_bind_ of − 13.19 kcal/mol, highlighting its potent binding energetics.

**Table 1:** Molecular docking and binding free energy results

Compounds	Docking score kcal/mol	Binding free energy kcal/mol
Olmutinib	− 4.93	− 12,13
H	− 6.62	− 11.56
Br	− 5.47	− 12.56
Cl	− 5.81	− 10.26
F	− 5.56	− 10.18
P-methoxy	− 4.87	− 11.63
Nitro	− 6.31	− 13.19
Methyl	− 6.95	− 12.86

The bromine (Br) derivative presented a balanced profile, with a docking score of − 5.47 kcal/mol and a ΔGbind of − 12.56 kcal/mol, underpinned by robust hydrophobic interactions that contribute to a stable ligand-receptor complex. In contrast, the hydrogen (H) derivative, despite a promising docking score of − 6.62 kcal/mol, recorded a less favourable ΔGbind of − 11.56 kcal/mol compared to olmutinib. The fluorine (F) and chlorine (Cl) derivatives, with docking scores of − 5.56 kcal/mol and -5.81 kcal/mol, respectively, showed moderate binding potential but weaker ΔGbind values (− 10.18 and − 10.26 kcal/mol), indicating fewer effective interactions with EGFR. Collectively, the methyl, nitro, and bromine derivatives outperformed olmutinib, positioning them as promising leads for further development into next-generation EGFR inhibitors tailored for breast cancer treatment.

### Protein–ligand interaction analysis

**Fig. 5:**
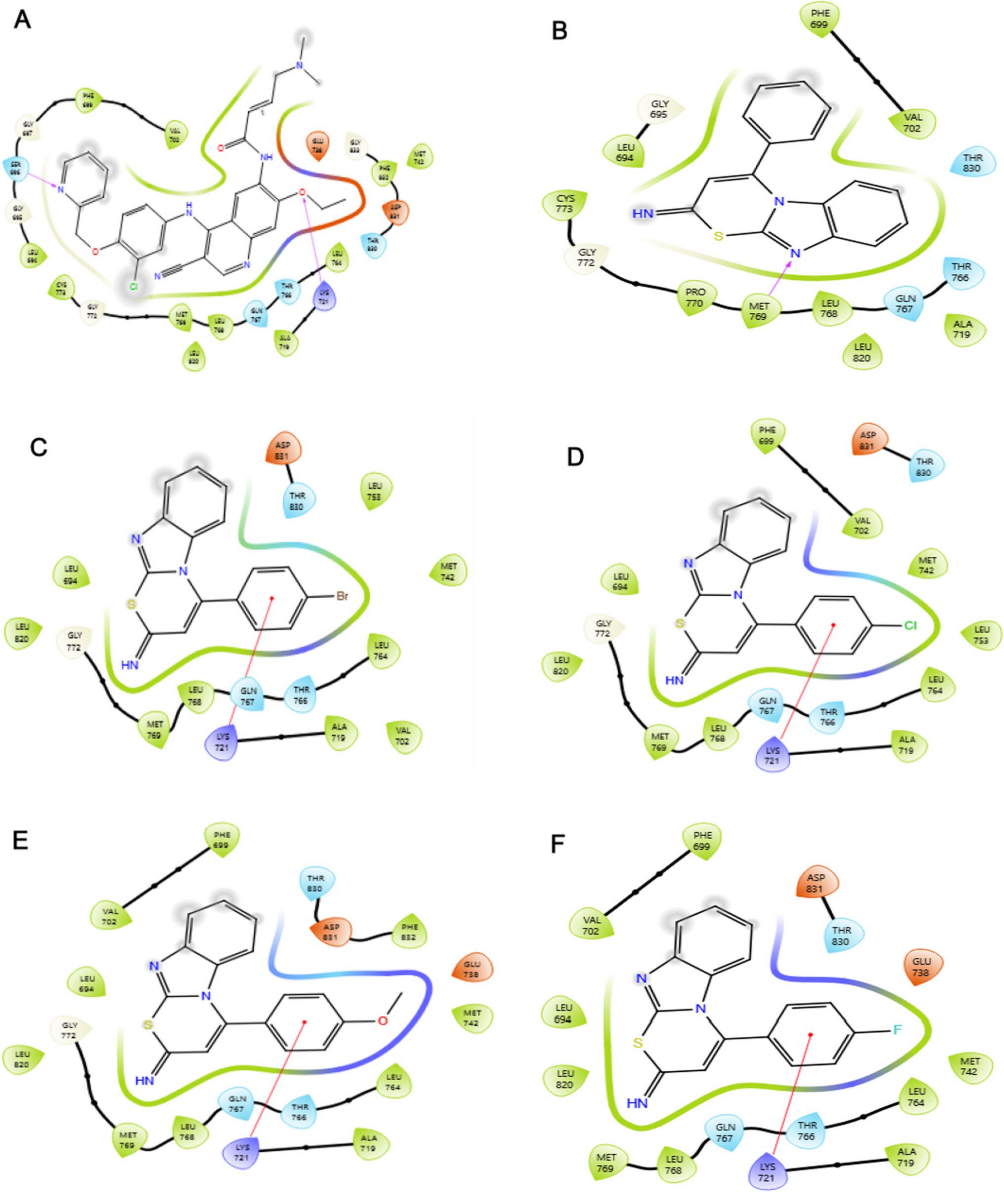

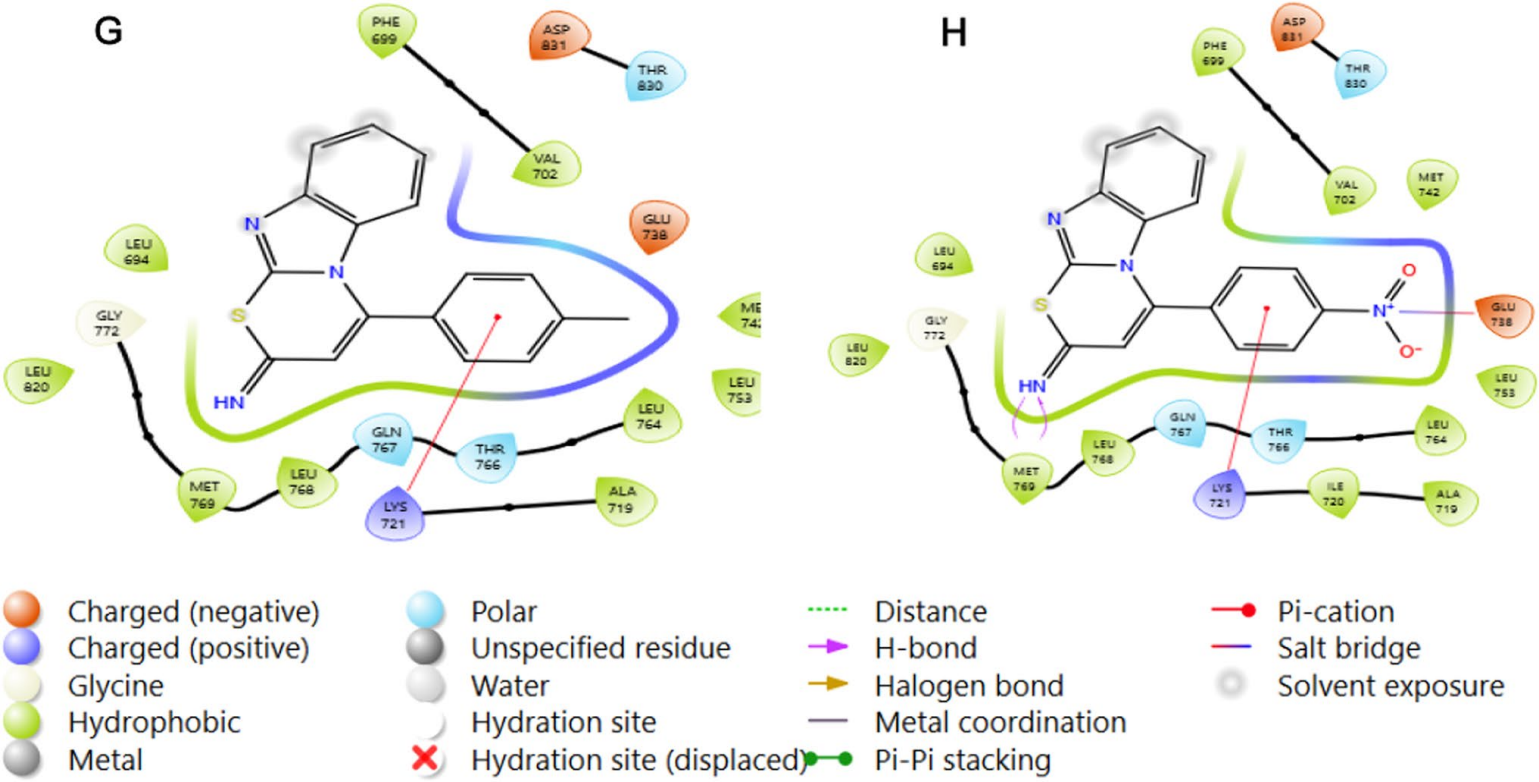
Protein–Ligand Interaction of **A** Olmutinib, **B** Compound H, **C** Compound Br, **D** Compound Cl, **E** Compound P-methoxy, **F** Compound F, **G** Compound methyl, and **H** Compound nitro with amino acid residues of EGFR (1M17). The compounds form hydrogen bonds primarily with MET769, while π-cation interactions are observed with LYS721. Hydrophobic contacts with residues such as LEU768, THR766, and PHE699 further stabilize the complex

Molecular docking provides critical insights into the interactions between ligands and the EGFR protein (PDB ID: 1M17), highlighting key binding features such as hydrogen bonds, pi-pi stacking, hydrophobic contacts, electrostatic interactions, and polar contacts that influence binding affinity and stability (Kitchen et al. 2004; Pan et al. 2022). These interactions, visualized across the multi-panel Fig. 5, reveal a consistent engagement of core residues, including LEU768, MET769, GLN767, and LYS721, across all nine thiazine derivatives and the reference compound Olmutinib, underscoring a shared binding scaffold within the EGFR active site.

Olmutinib exhibits moderate binding, characterized by hydrophobic interactions with LEU768 and PHE699, supplemented by a single hydrogen bond with MET769, reflecting a balanced but less optimized interaction profile. The thiazine derivatives, however, display varying degrees of enhancement. Compound h mirrors olmutinib's pattern, with hydrophobic contacts to PHE699 and LEU768, reinforced by a hydrogen bond with MET769; however, its more compact orientation suggests an improved fit. Compound br enhances this profile with additional hydrophobic interactions involving MET742 and MET769, as well as polar contacts with LYS721 and GLN767, indicating greater stability. In contrast, compound cl relies heavily on hydrophobic engagements with PHE699 and LEU768, with weaker polar interactions at LYS721 and potential dipole contributions from GLN767 and THR766, lacking strong hydrogen bonds. Compound p-methoxy and compound f show similar trends, with stable hydrophobic embedding via GLU738, THR766, and LEU768 (p-methoxy) and MET769, LEU768, and PHE699 (f), respectively, alongside weaker polar interactions with LYS721 and GLN767. These interactions suggest a dipole potential but fall short of robust electrostatic bonding. Compound methyl stands out due to its extensive hydrophobic contacts with MET769, LEU768, and PHE699, complemented by a strong hydrogen bond with MET769, which aligns with its superior docking and MM-GBSA scores. Compound nitro excels further, featuring a rich interaction network that includes a hydrogen bond to MET769, strong ionic interactions between the oxygen atoms of its nitro group and GLU738 and LYS721, as well as hydrophobic contacts with PHE699, CYS773, and LEU768, which reflects its exceptional binding energetics.

The standout performers, methyl, br, and nitro, distinguish themselves through more diverse and robust interaction profiles, including stronger hydrogen bonds and electrostatic contributions, which correlate with their favourable docking scores and binding free energies. This enhanced complementarity with the EGFR active site positions these derivatives as promising leads for further optimization, while the varying interaction strengths across the series highlight opportunities for structural refinement to boost efficacy.

All the thiazine compounds analysed were found to form a pi-cation interaction with LYS721, a positively charged amino acid situated in the ATP-binding region of EGFR. According to Palanivel et al. (2021), this residue plays a crucial role in kinase inhibition, as its interaction with inhibitors helps stabilize ligand binding and prevents ATP from entering the catalytic site. Similarly, Türkmenoğlu (2022) emphasized that pi-cation interaction with LYS721 is a recurring feature among potent EGFR inhibitors, contributing directly to their inhibitory activity.

### ADME analysis

The ADME properties of nine thiazine derivatives and the reference compound olmutinib were predicted using the QikProp module, providing a comprehensive assessment of their absorption, distribution, metabolism, and excretion profiles (Schrödinger 2023b). All compounds adhered to the Lipinski Rule of Five (Ro5), demonstrating no violations and confirming their suitability for oral drug-likeness, characterized by molecular weights below 500 g/mol, balanced lipophilicity (Log P < 5), and favourable hydrogen bond donor and acceptor counts that enhance absorption and permeability, as detailed in Table 2. Olmutinib, despite being an approved drug, exhibited limitations due to poor solubility, an elevated solvent-accessible surface area (SASA) of 831 Å^2^, and reduced permeability, which is consistent with its known pharmacokinetic challenges. In contrast, the thiazine derivatives exhibited more compact structures with SASA values ranging from 500 to 540 Å^2^, facilitating improved diffusion across membranes.

**Table 2:** ADME results

Properties	Optimum value	H	Nitro	Methyl	F	Br	P-methoxy	Cl	Olmutinib
MW	< 500	277	322	291	295	356	307	312	487
CNS	− 2 (Inactive) + 2 (Active)	0	0	− 2	0	0	0	0	1
Dipole	1–12.5	5.2	2.1	5.8	2.8	3.4	4.9	3	2.9
SASA	300–1000	503	543	534	512	531	524	521	831
DonorHB	0–6	1	1	1	1	1	1	1	2
AccptHB	2–20	2.5	3.5	2.5	2.5	2.5	3.5	2.5	8.5
QPlogPo/w	− 2–6.5	3.6	2.9	3.9	3.9	4.2	3.7	4.1	4.5
QPlogS	− 6.5–0.5	− 4.4	− 4.6	− 5.0	− 4.8	− 5.3	− 4.4	− 5.0	− 6.2
QPPCaco	< 25 poor, > 500 great	1556	185	1557	1562	1562	1597	1593	320
QPlogBB	− 3–1.2	− 0.195	− 1.19	− 0.220	− 0.088	− 0.027	− 0.230	− 0.015	− 0.358
QPP-MDCK	< 25 poor, > 500 great	1410	141	1411	2558	3757	1450	3560	277
QPlogKhsa	− 1.5–1.5	0.39	0.35	0.54	0.43	0.53	0.39	0.50	0.73
Metab	1–8	1	2	2	1	1	2	1	3
%HOA	< 25% poor, > 80% high	100	84.8	100	100	100	100	100	100
PSA	7–200	41.4	86.2	41.3	41.3	41.3	49.8	41.3	84.7
RO3		0	0	0	0	0	0	0	1
RO5		0	0	0	0	0	0	0	0

MW (Molecular Weight), CNS (Central Nervous System activity/potential), Dipole (Dipole moment), SASA (Solvent Accessible Surface Area), DonorHB (Hydrogen Bond Donors), AccptHB (Hydrogen Bond Acceptors), PSA (Polar Surface Area). Lipophilicity and solubility: QPlogPo/w (Predicted octanol/water partition coefficient), QPlogS (Predicted aqueous solubility). Permeability and absorption: QPPCaco nm·s⁻^1^ (Predicted Caco-2 cell permeability), QPlogBB (Predicted blood–brain barrier permeability), QPP-MDCK nm·s⁻^1^ (Predicted MDCK cell permeability), %HOA (Percent Human Oral Absorption). Metabolism and binding: Metab (Number of metabolic sites), QPlogKhsa (Predicted binding to human serum albumin). Rule-based evaluations: RO3 (Rule of Three), RO5 (Rule of Five) (Kumar and Ayyannan 2023.

Compounds h, methyl, br, f, p-methoxy, and cl demonstrated exceptional membrane permeability, with Caco-2 and MDCK values exceeding 1400 and 100% predicted human oral absorption, markedly outperforming the lower permeability observed in nitro and olmutinib. The predicted human oral absorption (%HOA) of 100% is an estimate provided by QikProp based on calculated permeability and physicochemical properties; it does not represent experimentally measured absorption. Notably, nitro distinguished itself by exhibiting poor blood–brain barrier penetration and reduced permeability, although this is offset by its high binding affinity, suggesting a selective profile. Most thiazine derivatives, excluding nitro and olmutinib, maintained lower polar surface areas (PSA), enhancing permeability potential. Additionally, these derivatives exhibited greater metabolic stability, with predicted metabolic sites limited to 1–2, compared to olmutinib, which has three. All thiazine compounds complied with Jorgensen's Rule of Three (Ro3), whereas olmutinib breached one criterion due to its suboptimal Caco-2 permeability of 320.

Overall, the thiazine derivatives, particularly h, methyl, and cl, showcased superior pharmacokinetic characteristics compared to olmutinib, including enhanced permeability, oral absorption, and metabolic stability. These attributes position them as promising candidates for further optimization as orally bioavailable EGFR inhibitors.

However, it is essential to note that ADMET predictions are inherently dependent on the quality and scope of the experimental data used to train the predictive models and therefore serve as theoretical estimations rather than definitive pharmacokinetic measurements (Kesharwani et al. 2020). Similarly, molecular docking scores may not fully capture real binding energetics due to simplified scoring functions that overlook entropic and solvation effects as well as non-classical interactions such as halogen and guanidine-arginine bonding (Sethi et al. 2020). To mitigate these limitations, molecular dynamics (MD) simulations and MM-GBSA binding energy analyses were performed, confirming the dynamic stability and favourable binding behaviour of the top thiazine–EGFR complexes under physiological conditions.

### Molecular dynamics simulation and post-MD analysis

**Fig. 6:**
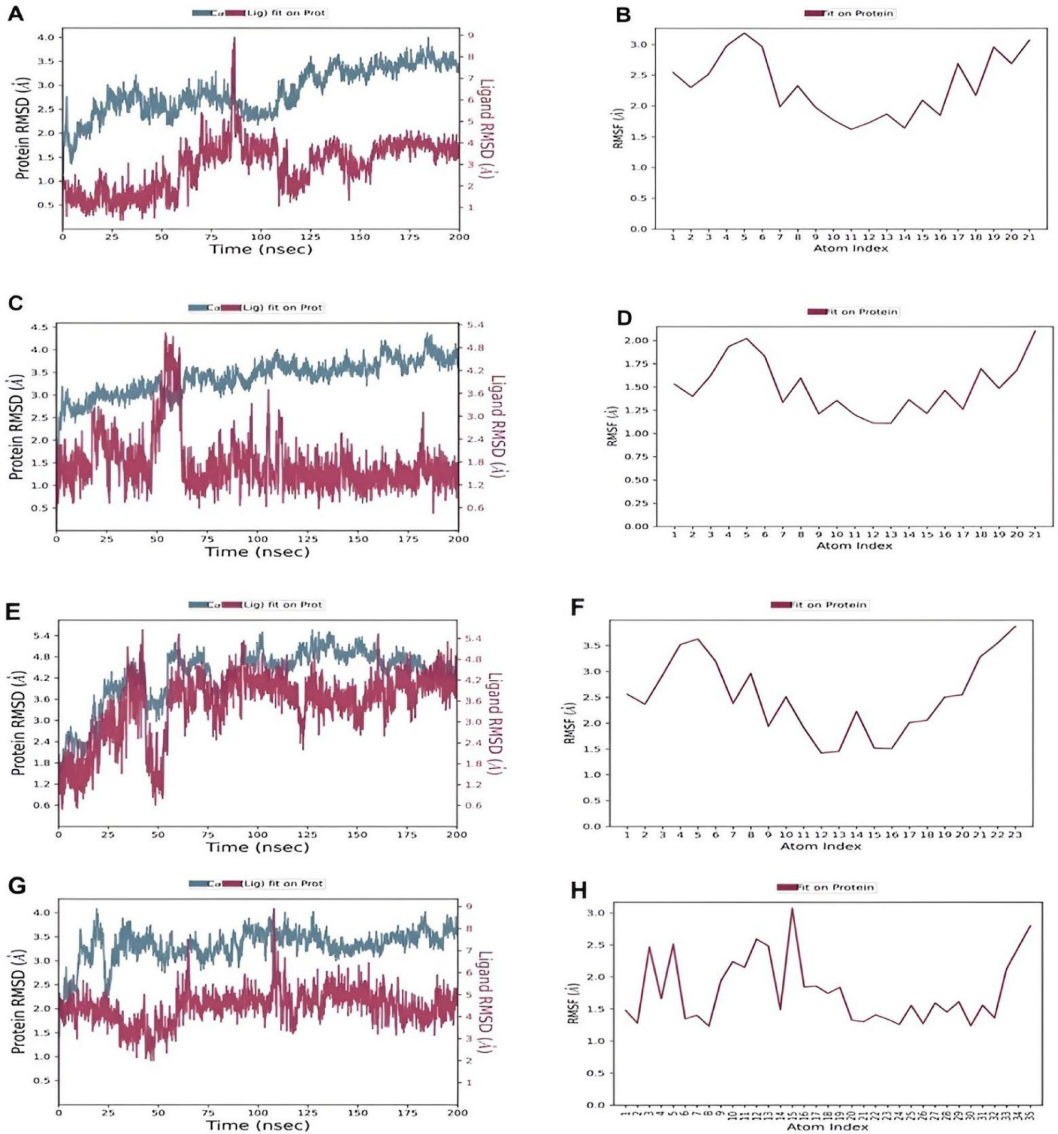
Protein–ligand RMSD and RMSF for **A**, **B** Methyl thiazine derivative, **C**, **D** Bromo thiazine derivative, **E**, **F** Nitro thiazine derivative, **G**, **H** Olmutinib over 200 ns

Molecular dynamics (MD) simulations were conducted to evaluate the stability and dynamic behaviour of ligand-EGFR complexes over a 200 ns timeframe, utilizing key metrics such as Root Mean Square Deviation (RMSD), Root Mean Square Fluctuation (RMSF), Solvent-Accessible Surface Area (SASA), and Radius of Gyration (rGyr) (Adcock and McCammon 2006). RMSD measures the overall deviation from the initial structure, while RMSF assesses residue-specific flexibility, offering valuable insights into binding stability. Based on prior docking and binding energy analyses, the top-performing thiazine derivatives methyl, bromo, and nitro were selected for detailed examination in the main manuscript, with their RMSD and RMSF profiles presented in Fig. 6A–H. Data for the remaining derivatives (f, p-methoxy, cl, h) are provided in Supplementary Figs. S1 and S2 to support a comprehensive analysis.

The RMSD profiles revealed notable differences in stability among the compounds. From Fig. 6 and Table 3, the hydrogen (h) derivative exhibited the lowest RMSD at 1.18 Å, followed by br at 1.77 Å, fluorine (f) and chlorine (cl) at 2.59 Å and 2.80 Å, respectively, and methyl at 2.91 Å, indicating minimal structural deviation and robust maintenance of their initial binding poses within the EGFR active site. olmutinib, with an RMSD of 4.47 Å, displayed significant instability and fluctuating binding, likely due to a suboptimal fit. Nitro, with an RMSD of 3.47 Å, showed moderate stability, aligning with its strong initial binding energy but suggesting dynamic variability over time. The nitro derivative showed moderate RMSD but a highly favourable MM-GBSA binding energy (-55.19 kJ/mol), indicating that despite some conformational flexibility, strong interactions with key residues were maintained. In contrast, compound h exhibited the lowest RMSD (1.18 Å), reflecting excellent structural stability, but its binding energy was lower than the reference, suggesting weaker thermodynamic interactions. These results illustrate that structural stability alone does not guarantee strong binding, and that combining RMSD and MM-GBSA analyses provides a more complete understanding of ligand performance.

**Table 3:** Summary of molecular dynamics simulation results

Compounds	RMSD (Å)	RMSF (Å)	rGyr (Å)	SASA(Å^2^)
F	2.59	2.24	3.57	58.10
P-methoxy	3.95	3.20	3.98	67.10
Br	1.77	1.50	3.66	49.20
Cl	2.80	1.39	3.57	94.10
Nitro	3.47	2.52	3.74	55.20
H	1.18	0.80	3.59	73.30
Methyl	2.91	2.33	3.88	53.00
Olmutinib	4.47	1.78	5.75	285.0

The compounds that maintained low RMSD values (< 2 Å) demonstrated strong and stable binding within the EGFR active site, exhibiting minimal structural deviation throughout the simulation. In line with the findings of Agarwal et al. (2022), who observed stable RMSD profiles for natural product inhibitors over a 100-ns trajectory, these results indicate that the ligands remained consistently bound to the protein throughout the entire simulation period.

RMSF analysis further illuminated these trends, with br at 1.50 Å and h at 0.80 Å reflecting reduced residue flexibility in the active site, indicative of tighter binding. In agreement with this, Emami et al. (2022) reported that lower RMSF values are associated with reduced atomic fluctuations and increased structural compactness of the protein, which collectively indicate greater stability of the protein–ligand complex. In contrast, olmutinib's RMSF of 1.78 Å and nitro's 2.52 Å indicated greater local fluctuations, suggesting less conformational stability (Van Der Spoel et al. 2005).

**Fig. 7:**
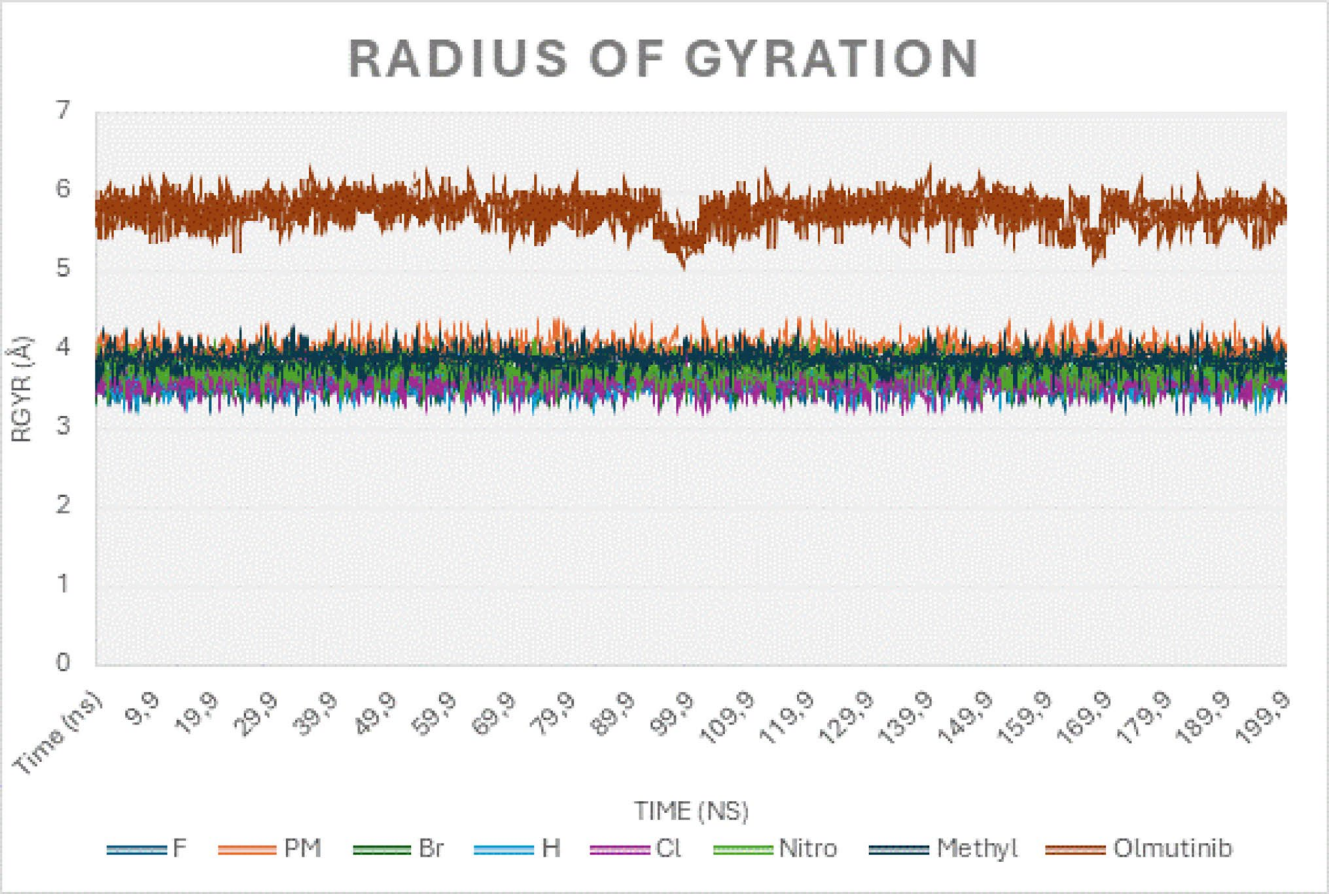
Radius of gyration dynamics over 200 ns molecular dynamics simulation

SASA and rGyr (Table 3) analyses provided more profound insights into the compactness of the complexes. Br and methyl recorded the lowest SASA values at 49.20 Å^2^ and 53.00 Å^2^, respectively, signifying deep embedding within the EGFR pocket and enhanced stability. At the same time, olmutinib's SASA of 285.0 Å^2^ suggested a more exposed, less tightly bound conformation. Nitro's SASA of 55.20 Å^2^ indicated a moderately stable binding mode, although less optimal than that of the top performers. The rGyr values, as shown in Table 3 for br (3.66 Å), h (3.59 Å), and methyl (3.88 Å), were lower and more stable, reflecting compact and conformationally intact complexes. In contrast, olmutinib's rGyr of 5.75 Å and p-methoxy's 3.98 Å correlated with less stable binding dynamics. The alignment of SASA and rGyr data highlights the superior dynamic stability of methyl, br, and nitro groups, with the thiazine derivatives collectively demonstrating greater resilience and compactness compared to Olmutinib, as illustrated in Fig. 7 (Bakan et al. 2011). The thiazine compounds maintained stable structural compactness throughout the simulation, exhibiting only minor fluctuations compared to olmutinib, as shown in Fig. 7. This stability indicates strong binding and minimal conformational changes, suggesting robust and durable interactions with the EGFR protein. The consistently low radius of gyration (rGyr) values (below 4 Å) observed for the thiazine EGFR complexes further confirm a compact and stable conformation. These findings are consistent with previous reports, such as Balkrishna et al. (2024), who demonstrated that lower rGyr values correlate with enhanced protein–ligand stability, as observed in the luteolin HSP90 complex.

These results underscore the potential of methyl, br, and nitro as lead candidates, supported by their stable binding profiles. At the same time, supplementary data for the other derivatives offer a foundation for future refinement.

### Protein–ligand contact analysis

**Fig. 8:**
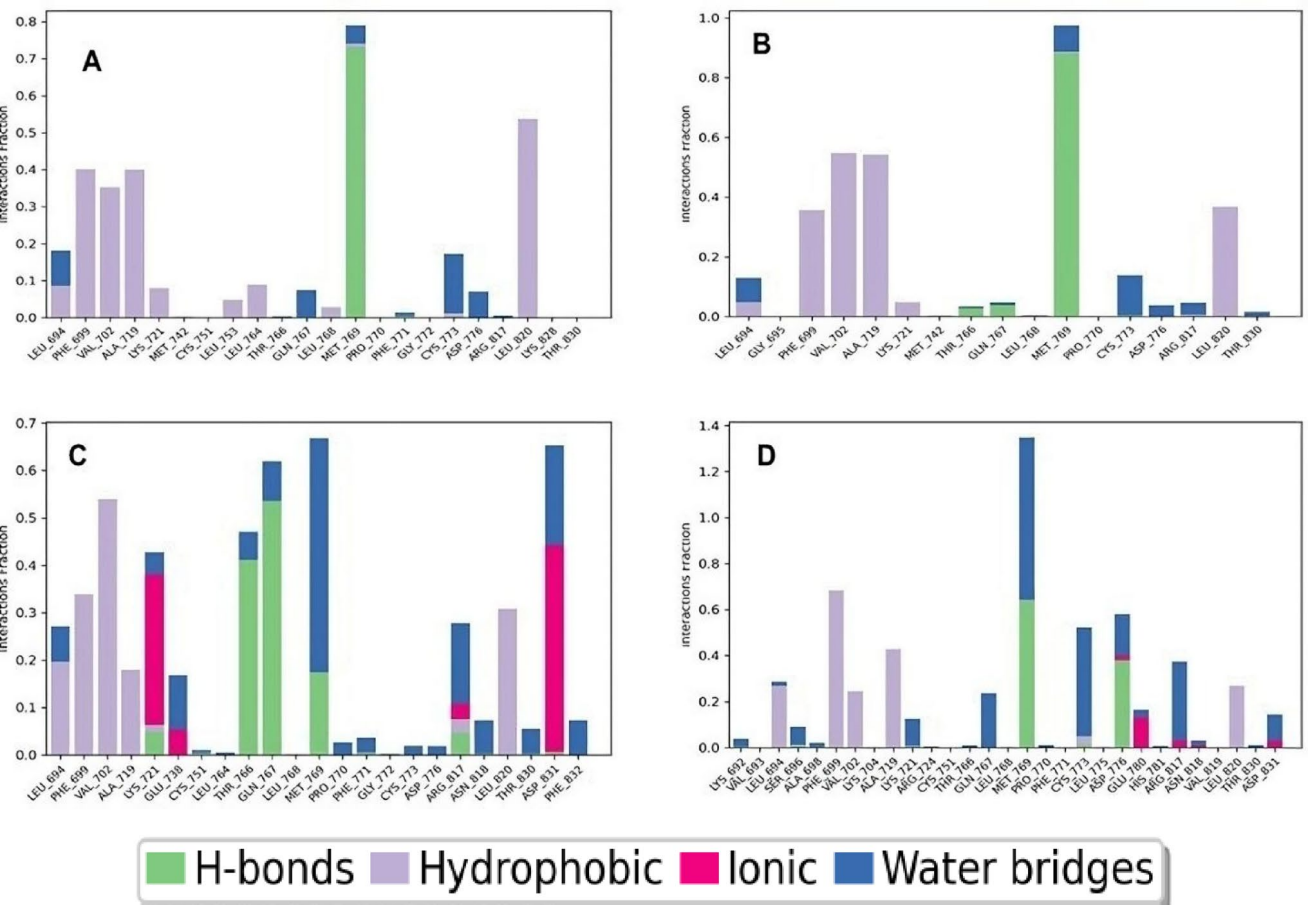
Protein–ligand contact for **A** Methyl derivative, **B** Bromo derivatives, **C** Nitro derivative and **D** Olmutinib

The chemical nature of ligand–protein interactions within the EGFR active site was further elucidated, with hydrophobic interactions emerging as the dominant force, complemented by significant contributions from van der Waals forces and π-π stacking (Goodsell and Olson 1990). This analysis, illustrated in Fig. 8 for the top-performing thiazine derivatives methyl, bromo, and nitro, alongside the reference compound olmutinib, highlights their binding characteristics. Hydrophobic ligands, such as methyl and bromine, thrive when deeply embedded within the protein pocket, where low Solvent-Accessible Surface Area (SASA) values, such as 53.0 Å^2^ for methyl and 49.2 Å^2^ for bromine, correlate with enhanced binding stability and favorable interactions. This burial within the hydrophobic core highlights the crucial role of these interactions in stabilizing a ligand-EGFR complex.

In contrast, polar ligands like nitro and olmutinib rely on hydrogen bonds and electrostatic interactions to anchor their binding, as depicted in Fig. 8C, D. While nitro benefits from ionic interactions with residues such as GLU738 and LYS721, and olmutinib forms a hydrogen bond with MET769, their effectiveness depends on precise orientation within the binding site, with some tolerance for solvent exposure.

The hydrophobic residues VAL702, ALA719, and LEU820 identified in this study align with those reported as key contributors to ligand binding and stability within the EGFR kinase domain in previous research. Similarly, Moussaoui et al. (2024) highlighted the significance of these residues, showing that quinazoline derivatives maintained stable van der Waals and hydrophobic interactions with VAL702, ALA719, and LEU820 throughout molecular dynamics simulations, thereby enhancing ligand stability within the binding pocket. In addition to the stable hydrophobic contacts, MET769 consistently emerged as a key interacting residue, forming both hydrogen-bonding and hydrophobic interactions with several derivatives, including the h, br, and methyl compounds. This residue's persistent involvement across different complexes suggests its crucial role in stabilizing ligand binding within the EGFR active site and enhancing overall complex stability. This observation is further supported by Abdelgawad et al. (2016), who found that the N-1 atom of their pyrazolo[3,4-d]pyrimidine derivatives formed a hydrogen bond with Met769, effectively anchoring the ligand within the EGFR active site. The recurrence of this residue as a critical interaction hotspot across various scaffolds underscores its pivotal role in stabilizing ligand binding and maintaining complex integrity within the EGFR kinase domain.

The remaining derivatives, hydrogen (H), chlorine (Cl), fluorine (F), and p-methoxy, are presented in Supplementary Fig. S3, where the potential of p-methoxy as a hydrogen bond acceptor is undermined by its suboptimal orientation, leading to increased solvent exposure and weaker interactions. This comparative analysis highlights the superior binding profiles of methyl, bromo, and nitro, driven by their optimized hydrophobic and polar interactions, positioning them as promising leads for further development. For nitro, moderate flexibility did not compromise binding due to strong residue interactions, whereas h remained highly stable but engaged in comparatively weaker interactions, resulting in lower binding energy. At the same time, the supplementary data provides context for refining the less effective derivatives (Grosdidier et al. 2011).

Although single 200 ns MD trajectories were sufficient for the exploratory scope of this study, we acknowledge that full convergence analyses, including RMSD stability plots as well as independent MD replicates, are important for strengthening reliability. These extended analyses will be incorporated in future work as part of a more comprehensive validation.

### Toxicological profile

Pharmacokinetic and toxicological data are crucial to the rational design of effective medications, providing essential insights into the safety and viability of lead compounds in pharmaceutical research (Taft et al.). In this study, the toxicity profiles of the lead compounds were carefully evaluated in conjunction with their binding and dynamic behaviour. Table 4 below represents toxicity predictions of Olmutinib and the thiazine lead compounds. Genetic toxicology tests, such as the AMES assay, are commonly used to classify compounds as mutagenic, non-mutagenic, or inconclusive. Depending on the intended application and regulatory requirements, a mutagenic result can restrict further development, limit the compound's use, or prevent its commercialization (Zeiger 2023). In silico AMES predictions using pkCSM indicated that Olmutinib is non-mutagenic, suggesting a low risk of genetic toxicity (Suharsanti 2025; Pires et al. 2015).

**Table 4:** Toxicological data of the lead compounds

Compounds	AMES toxicity	hERG I inhibitor	hERG II inhibitor	Hepatotoxicity	Skin Sensitisation
Olmutinib	No	No	Yes	Yes	No
Methyl	Yes	No	Yes	No	No
Nitro	Yes	No	Yes	No	No
Br	Yes	No	Yes	No	No

In contrast, the thiazine derivatives, methyl, nitro, and br were predicted to be AMES-positive, indicating a potential risk of mutagenicity. Positive AMES results suggest that the compounds may induce point mutations, which could lead to genomic instability in these systems.

These findings do not invalidate the computational binding affinity results, but rather underscore the need for structural refinement before progressing toward drug-likeness. To mitigate this risk, several medicinal chemistry strategies can be explored, including substituting electron-withdrawing groups that maintain activity or applying prodrug approaches to reduce direct genotoxic interactions. Based on the current predictions, the methyl and bromo derivatives may represent more suitable starting points than the nitro derivative, provided mutagenicity can be successfully minimized. In addition, experimental validation, such as Ames assays and micronucleus tests, will be crucial in future work to confirm these computational alerts and guide the safe optimization of compounds.

Drug-induced hepatotoxicity encompasses both acute and chronic liver injury and remains a significant challenge in drug development, as no universally accepted primary screening methods exist for early identification of hepatotoxic compounds (Odhiambo et al. 2025). In this study, olmutinib was predicted to be hepatotoxic, whereas the thiazine derivatives were predicted to be non-hepatotoxic, suggesting a potential advantage in liver safety.

Cardiotoxicity, often assessed by hERG channel inhibition, is another key long-term safety concern, as it can lead to QT prolongation and arrhythmias (Walker et al. 1999). All compounds, including olmutinib, were predicted to inhibit hERG-II but not hERG-I, indicating a moderate risk of cardiotoxicity that should be monitored through in vitro hERG assays and in vivo cardiac studies. Future studies should include in vitro hERG patch-clamp assays to confirm these findings. Additionally, medicinal chemistry strategies, such as modifying substituents that may interact with the hERG channel, can be employed to reduce liability while retaining EGFR inhibitory activity. These steps will ensure that the selected lead compounds, particularly those with methyl and bromo substitutions, are safer for further development.

All compounds were predicted to be non-sensitizing to the skin, suggesting a low risk of dermatological toxicity. While the thiazine compounds addressed some of olmutinib's toxicity challenges, their potential mutagenicity poses a significant concern that would need to be addressed in further development.

## Conclusion

This study harnessed computational methodologies to assess the potential of nine thiazine-based derivatives as inhibitors of the epidermal growth factor receptor (EGFR) in breast cancer. Molecular docking and MM-GBSA binding energy calculations identified methyl, nitro, and bromine derivatives as standout performers, exhibiting superior binding affinities and more favourable interaction energies compared to the reference compound olmutinib. ADMET profiling further underscored the pharmacokinetic strengths of these thiazine compounds, revealing excellent oral absorption, favourable solubility, minimal central nervous system penetration, and full compliance with Lipinski's Rule of Five and Jorgensen's Rule of Three.

Molecular dynamics simulations provided robust evidence of the stability and compactness of the EGFR-ligand complexes, with methyl and bromine derivatives demonstrating the lowest RMSD, RMSF, SASA, and rGyr values, indicative of highly stable and deeply embedded binding configurations. In contrast, olmutinib and the p-methoxy derivative exhibited reduced stability and more exposed conformations, highlighting their relative limitations. Toxicological evaluation revealed that the lead compounds mitigated liver toxicity and skin sensitization risks, while retaining hERG-II inhibition potential and exhibiting potential mutagenicity concerns. Collectively, these results position methyl and bromine as the most promising candidates for lead optimization, leveraging their potent binding affinities, advantageous pharmacokinetic profiles, and stable dynamic behaviour. Thiazine derivatives thus emerge as a compelling scaffold for developing next-generation EGFR inhibitors.

EGFR can develop point mutations that render it resistant to existing tyrosine kinase inhibitors, particularly T790M, L858R, and exon 19 deletions, which alter the ATP-binding site and reduce inhibitor affinity (Cross et al. 2014; Wang et al. 2016). A study conducted by Teng et al. (2011) reported EGFR mutations, specifically exon 19 deletions and exon 21 missense (L858R) mutations, in 11.8% of Triple Negative Breast Cancer (TNBC) samples analysed. All mutations were heterozygous deletions, suggesting they are likely dominant and may contribute to tumour development.

In this study, we focused on wild-type EGFR to establish a baseline understanding of thiazine–EGFR interactions. Future work will expand to mutant EGFR forms, using selectivity docking and molecular dynamics simulations to assess whether thiazine compounds maintain effective binding and stability in the presence of resistance mutations. To complement these computational studies, experimental validation will be conducted, including in vitro EGFR kinase inhibition assays, cell viability/proliferation studies in EGFR-overexpressing breast cancer cell lines, Ames test for mutagenicity, hERG patch-clamp assays to assess cardiotoxicity, and microsomal stability studies to evaluate metabolic stability. These experiments will provide critical confirmation of the bioactivity, safety, and pharmacokinetic properties of the prioritized methyl and bromine-substituted thiazine derivatives, guiding further optimization of thiazine-based EGFR inhibitors.

## Supplementary Information

Below is the link to the electronic supplementary material.

Supplementary Material 1

## Data Availability

All data generated during this study are provided within the manuscript and the supplementary data. No additional raw datasets are available beyond what is presented.
