# Supplementary material for: Molecular insights into the bioactivity of H-thiazine compounds against breast cancer cells: a computational study
Source: In Silico Pharmacol. 2026 Jan 14;14(1):36. doi: 10.1007/s40203-025-00542-y (PMC12804472; doi:10.1007/s40203-025-00542-y)
Supplement: Supplementary file 1 — Supplementary Material 1 [file 40203_2025_542_MOESM1_ESM.docx]

**Supplementary material**

**Molecular Insights into the Bioactivity of H-Thiazine Compounds Against Breast Cancer Cells: A Computational Study.**

Lesego M. Mogoane^1^, Vincent A. Obakachi^1^, Penny P. Govender^1^, Krishna K. Govender^1*^

^1^Department of Chemical Sciences, University of Johannesburg, South Africa.

*Corresponding author email: [krishnag@uj.ac.za](mailto:krishnag@uj.ac.za)

**Supplementary SMILES Strings of All Thiazine Derivatives Table 1**

| **Compounds** | **SMILES** |
| --- | --- |
| H | N=C1Sc2nc3ccccc3n2C(=C1)c1ccccc1 |
| Br | N=C1C=C(n2c(S1)nc1ccccc21)c1ccc(Br)cc1 |
| Cl | N=C1C=C(n2c(S1)nc1ccccc21)c1ccc(Cl)cc1 |
| F | N=C1C=C(n2c(S1)nc1ccccc21)c1ccc(F)cc1 |
| P-methoxy | N=C1Sc2nc3ccccc3n2C(=C1)c1ccc(cc1)OC |
| Nitro | O=N(=O)c1ccc(cc1)C1=CC(=N)Sc2nc3ccccc3n21 |
| Methyl | N=C1Sc2nc3ccccc3n2C(=C1)c1ccc(C)cc1 |
| M-M1 | N=C1Sc2nc3ccccc3n2C(=C1)c1cc(ccc1)OC |
| M-M2 | N=C1Sc2nc3ccccc3n2C(=C1)c1cc(ccc1)OC |

**Supplementary Docking scores Table 2**

| **Compounds** | **Docking Score** |
| --- | --- |
| M-M1 | -4.775 |
| M-M2 | -4.775 |

**Supplementary RMSD Fig. S1**


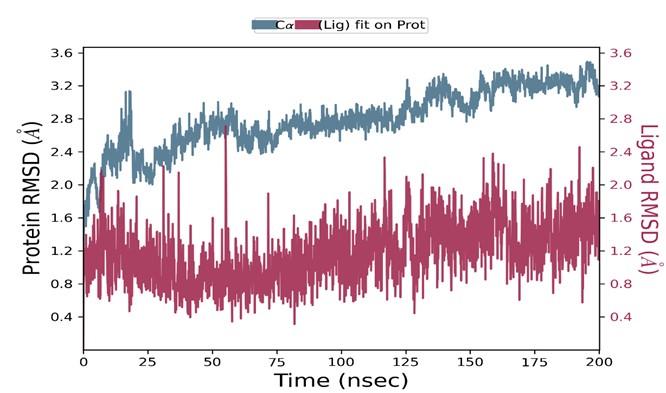


**Fig. S1 (a):** Protein-ligand RMSD for Compound H


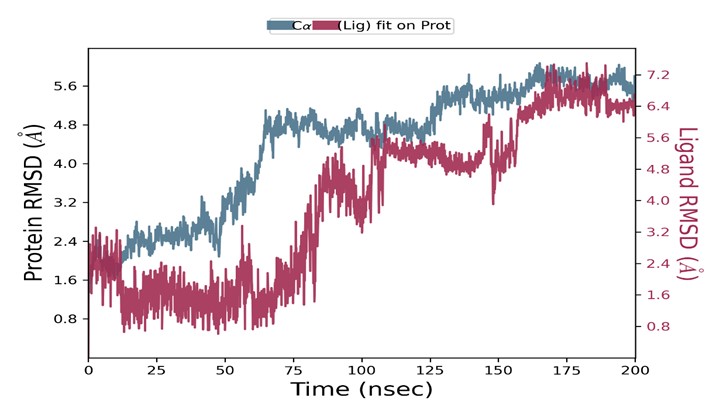


**Fig. S1 (b):** Protein-ligand RMSD for Compound PM.


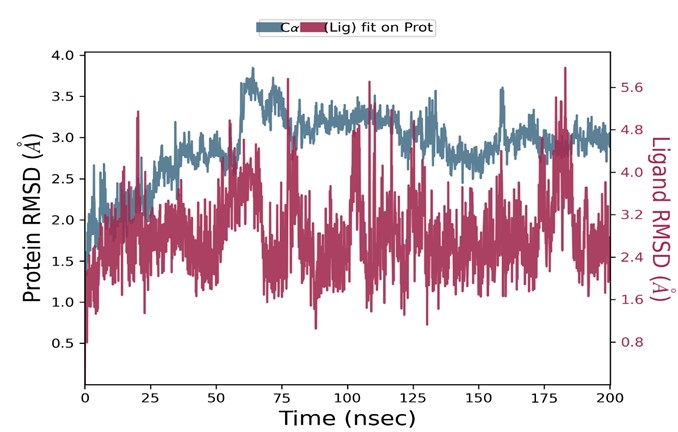


**Fig. S1 (c):** Protein-ligand RMSD for Compound Cl


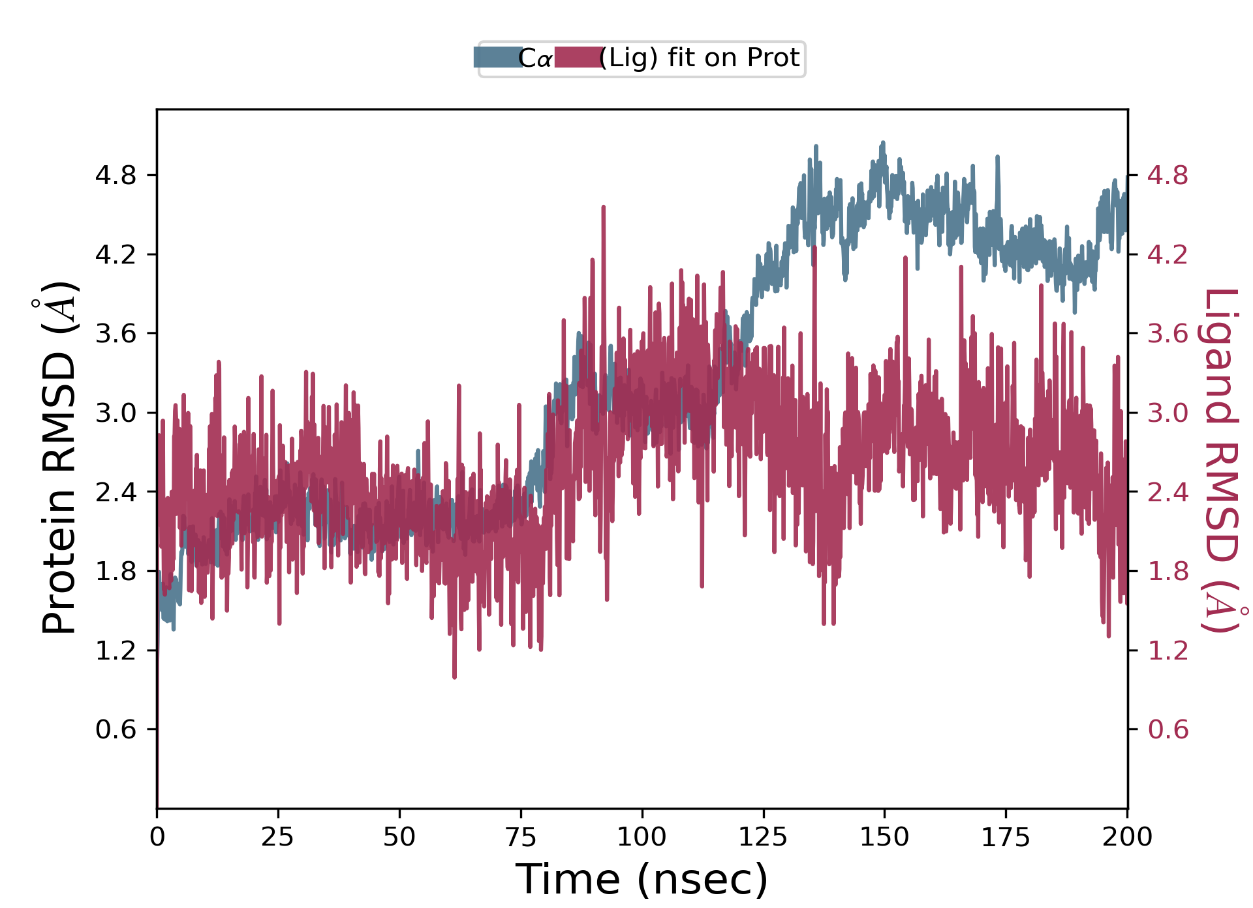


**Fig. S1 (d):** Protein-ligand RMSD for Compound F

**Supplementary RMSF Fig. S2**


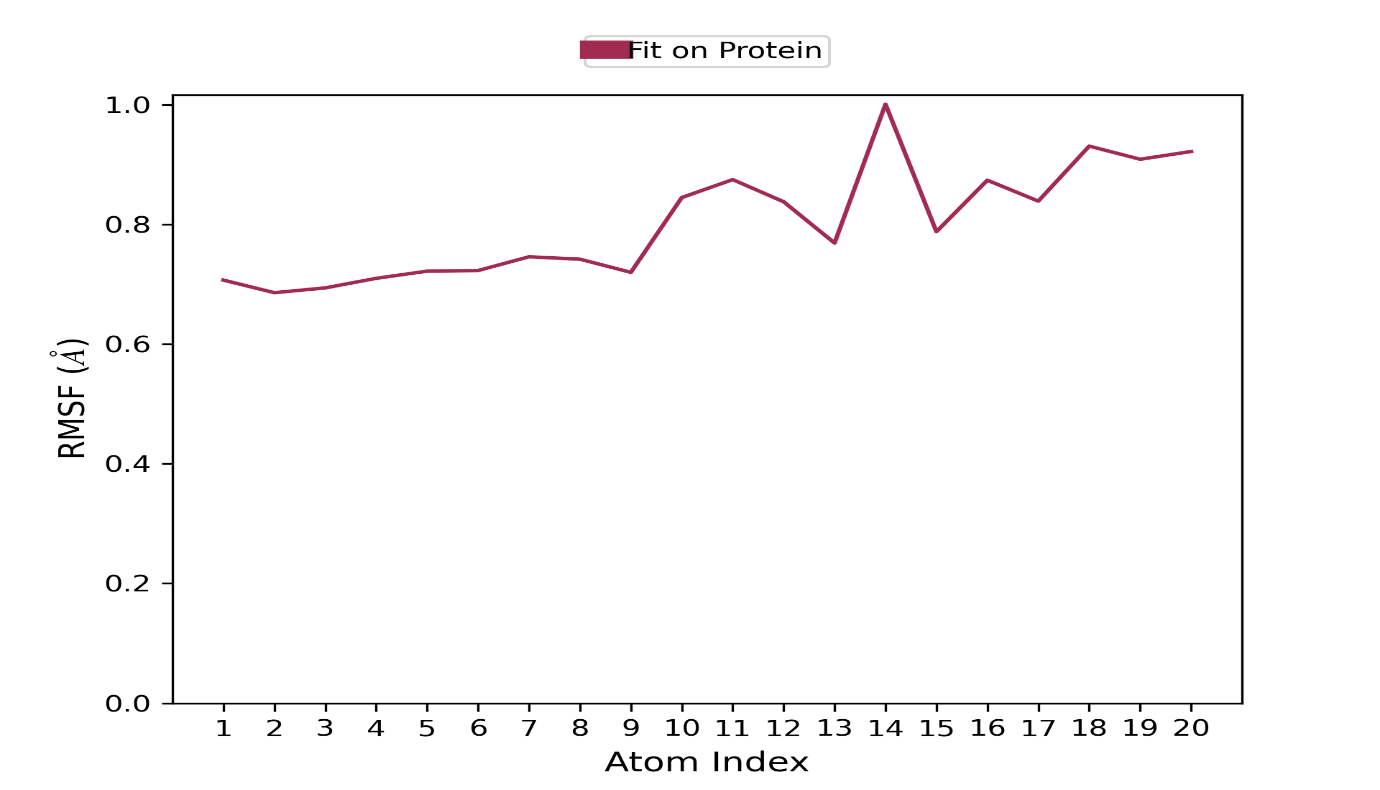


**Fig. S2 (a)**: RMSF graph of compound H over 200ns


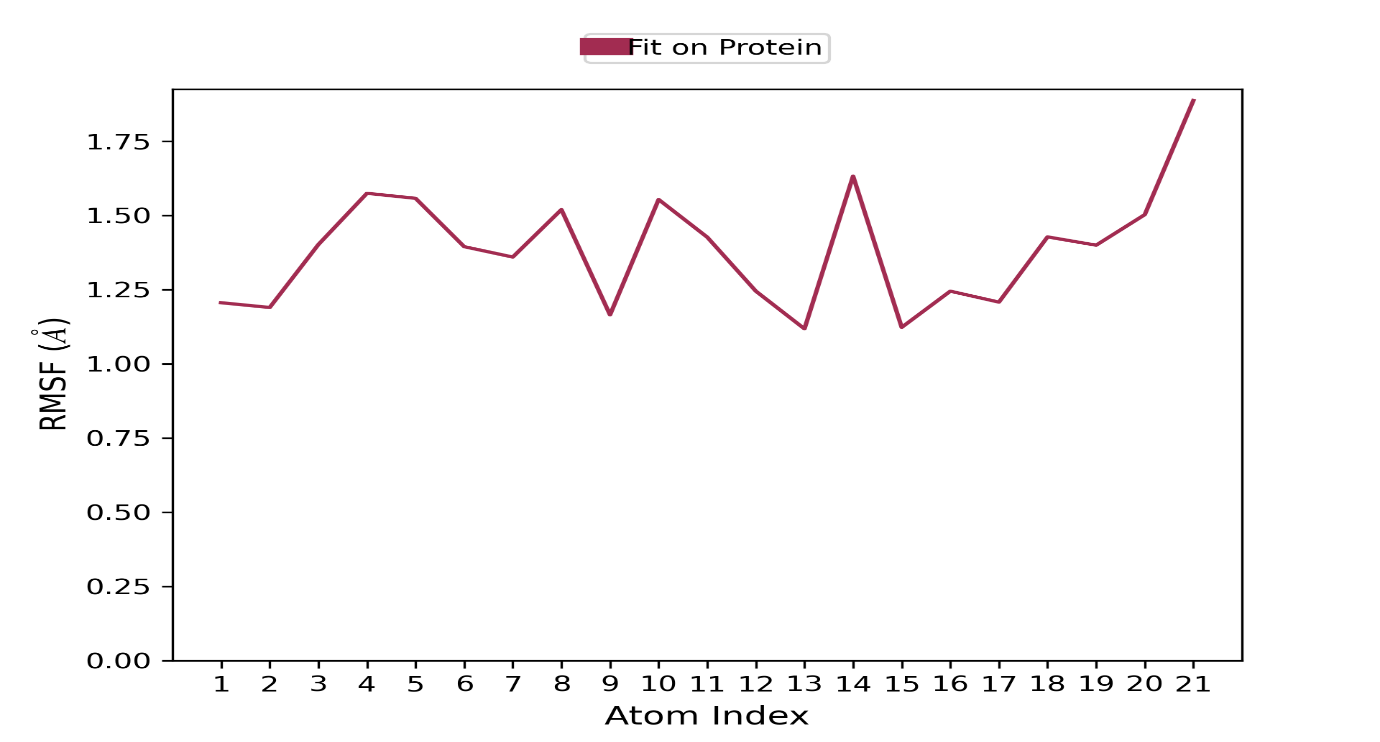


**Fig. S2 (b):** RMSF graph of compound Cl over 200ns.


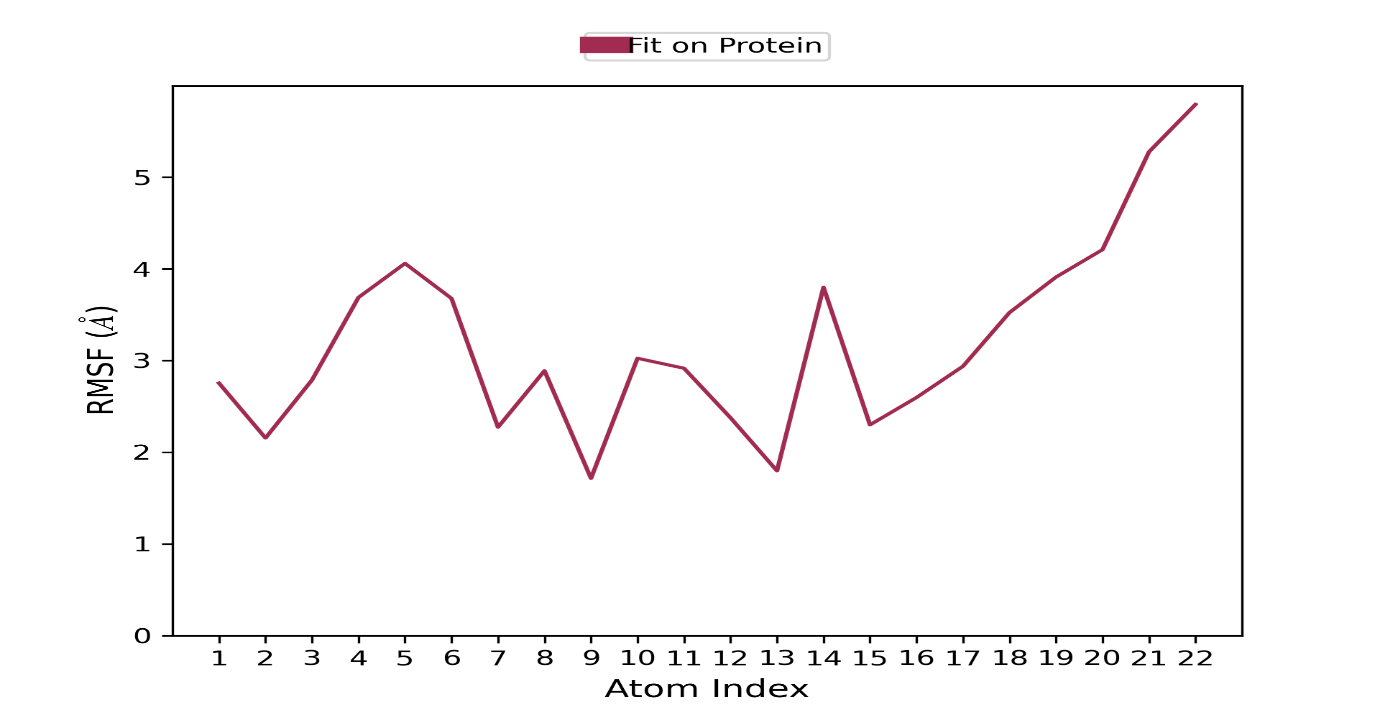


**Fig. S2 (c)**: RMSF graph of compound PM over 200ns.


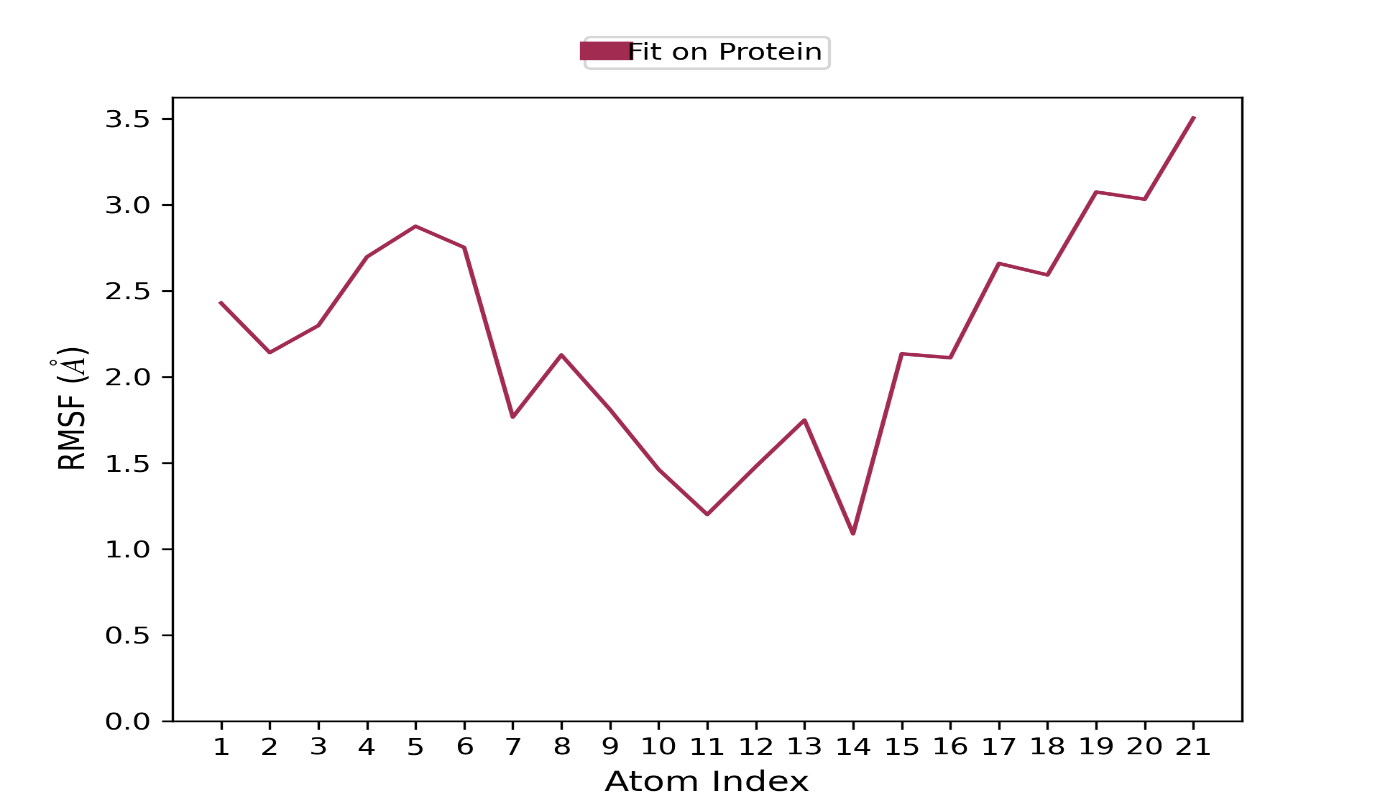


**Fig. S2 (d)**: RMSF graph of compound F over 200ns.

**Supplementary Fig. S3**


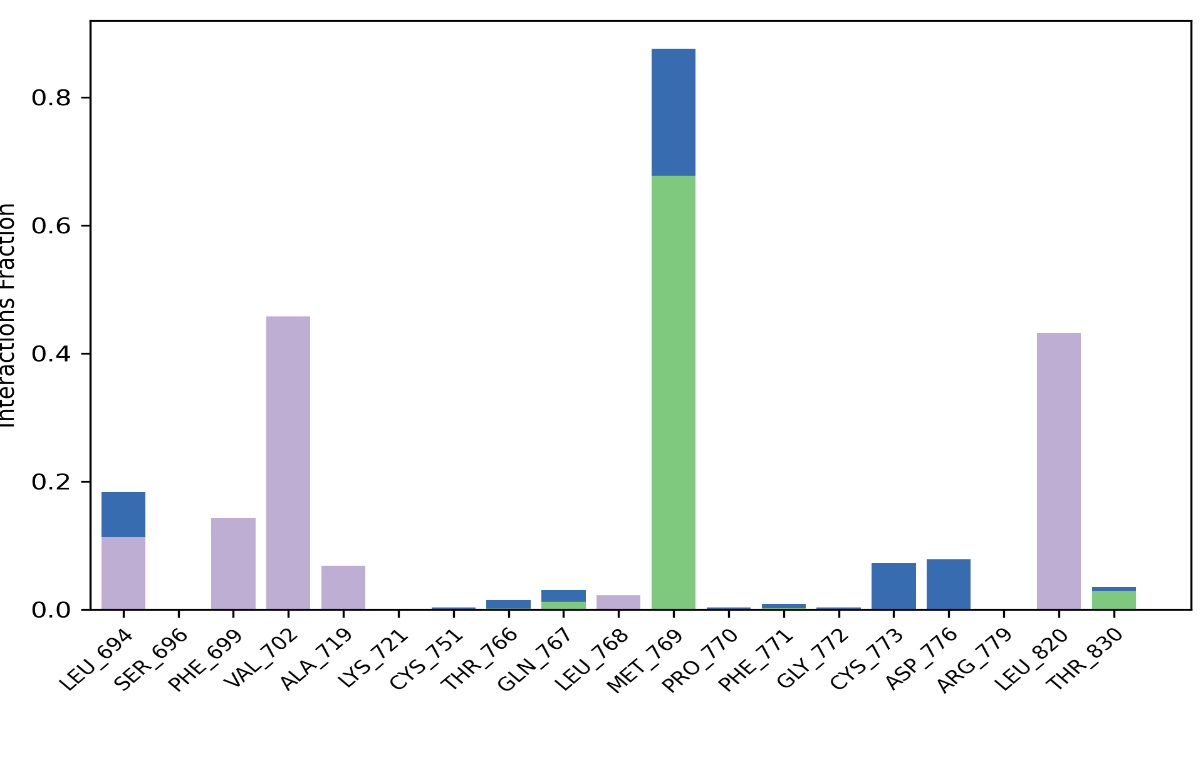


**Fig. S3 (a)**: Interaction between compound Cl and amino acid residues.


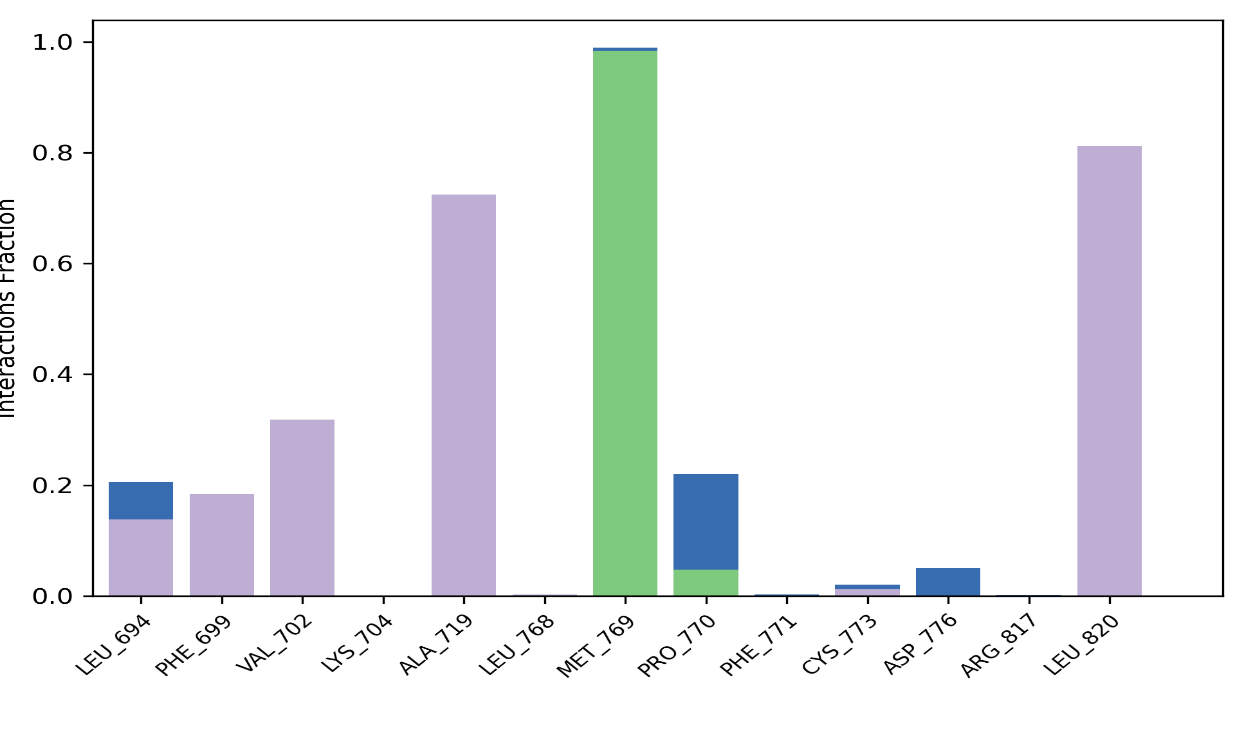


**Fig. S3 (b)**Interaction between compound H and amino acid residues.


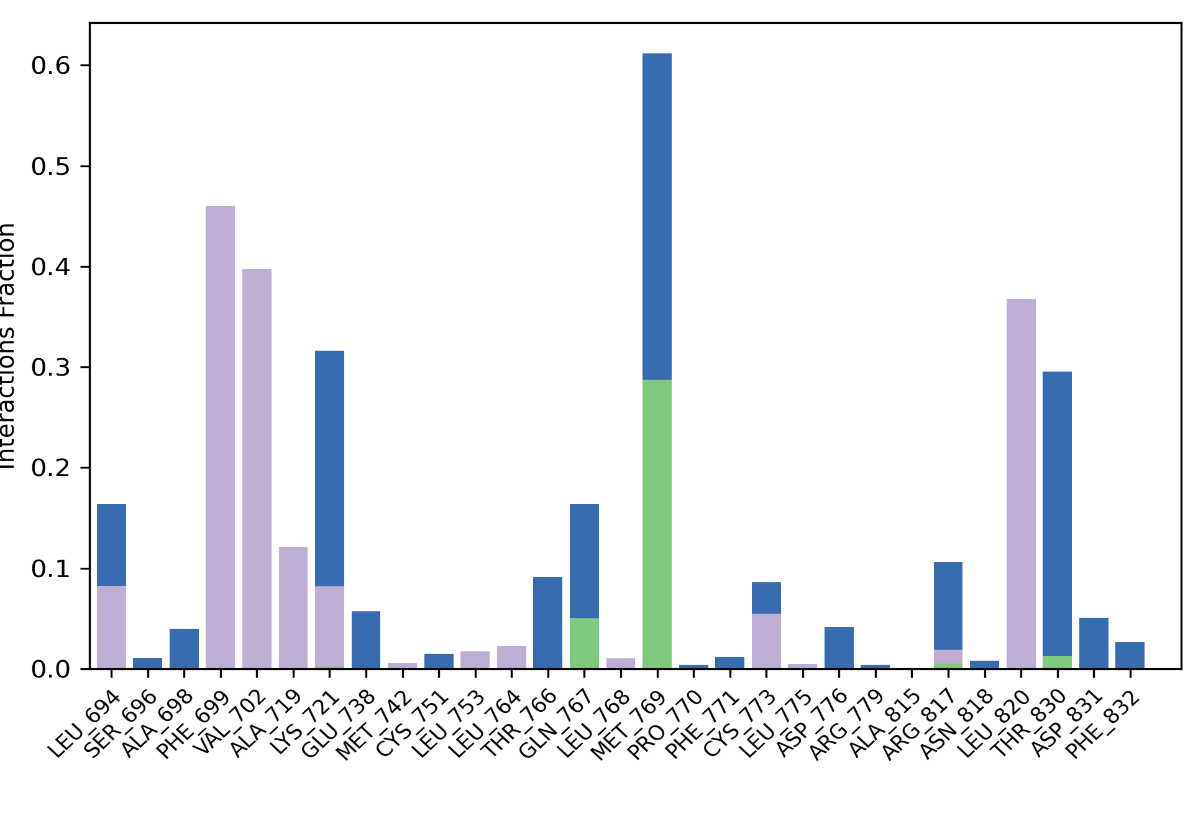


**Fig. S3(c)**: Interaction between PM and amino acid residues.

**
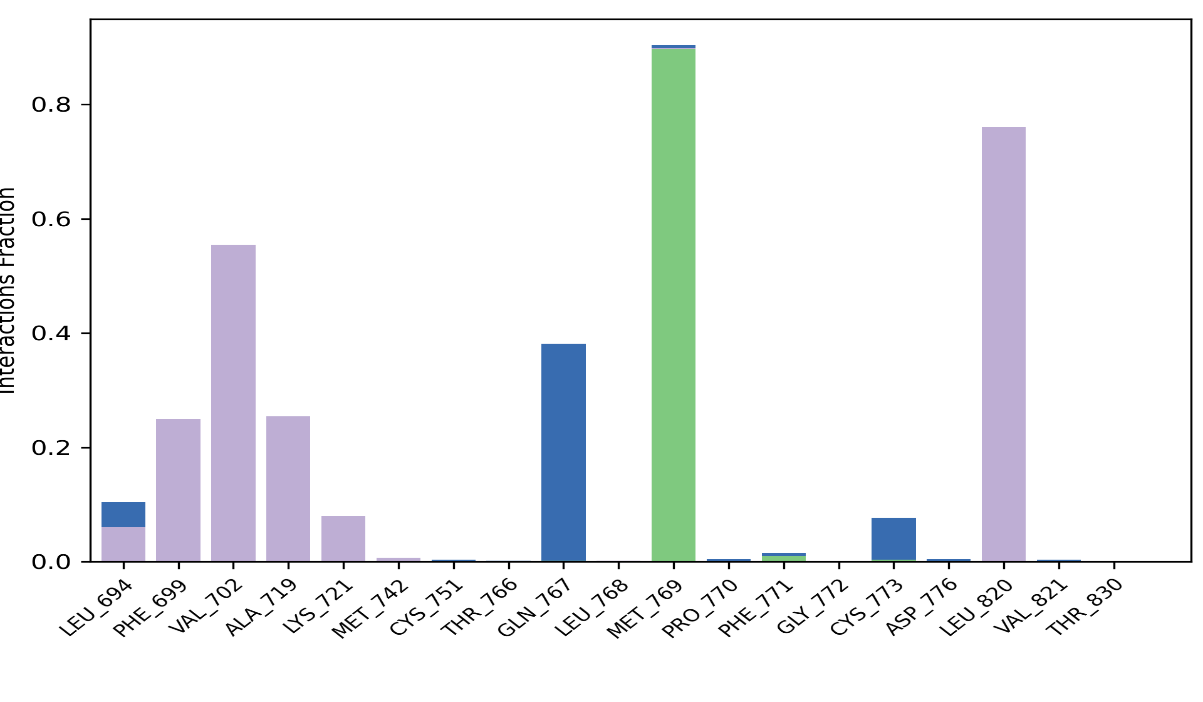
**

**Fig. S3(d)**: Interaction between F and amino acid residues.
